# Discovery of M Protease Inhibitors Encoded by SARS-CoV-2

**DOI:** 10.1128/AAC.00872-20

**Published:** 2020-08-20

**Authors:** Hui-Chen Hung, Yi-Yu Ke, Sheng Yu Huang, Peng-Nien Huang, Yu-An Kung, Teng-Yuan Chang, Kuei-Jung Yen, Tzu-Ting Peng, Shao-En Chang, Chin-Ting Huang, Ya-Ru Tsai, Szu-Huei Wu, Shiow-Ju Lee, Jiunn-Horng Lin, Bing-Sin Liu, Wang-Chou Sung, Shin-Ru Shih, Chiung-Tong Chen, John Tsu-An Hsu

**Affiliations:** a Institute of Biotechnology and Pharmaceutical Research, National Health Research Institutes, Miaoli, Taiwan; b Department of Biological Science and Technology, National Chiao Tung University, Hsinchu, Taiwan; c Department of Animal Technology Laboratories, Agricultural Technology Research Institute (ATRI), Miaoli, Taiwan; d Research Center for Emerging Viral Infections, Chang Gung University, Taoyuan, Taiwan; e Division of Infectious Diseases, Department of Pediatrics, Linkou Chang Gung Memorial Hospital, Taoyuan, Taiwan; f Department of Laboratory Medicine, Linkou Chang Gung Memorial Hospital, Taoyuan, Taiwan; g National Institute of Infectious Diseases and Vaccinology, National Health Research Institutes, Miaoli, Taiwan

**Keywords:** COVID-19, SARS-CoV-2, M^pro^, antiviral research, GC376, M protease

## Abstract

The coronavirus (CoV) disease 2019 (COVID-19) pandemic caused by severe acute respiratory syndrome CoV-2 (SARS-CoV-2) is a health threat worldwide. Viral main protease (M^pro^, also called 3C‐like protease [3CL^pro^]) is a therapeutic target for drug discovery. Herein, we report that GC376, a broad-spectrum inhibitor targeting M^pro^ in the picornavirus-like supercluster, is a potent inhibitor for the M^pro^ encoded by SARS-CoV-2, with a half-maximum inhibitory concentration (IC_50_) of 26.4 ± 1.1 nM. In this study, we also show that GC376 inhibits SARS-CoV-2 replication with a half-maximum effective concentration (EC_50_) of 0.91 ± 0.03 μM. Only a small portion of SARS-CoV-2 M^pro^ was covalently modified in the excess of GC376 as evaluated by mass spectrometry analysis, indicating that improved inhibitors are needed. Subsequently, molecular docking analysis revealed that the recognition and binding groups of GC376 within the active site of SARS-CoV-2 M^pro^ provide important new information for the optimization of GC376. Given that sufficient safety and efficacy data are available for GC376 as an investigational veterinary drug, expedited development of GC376, or its optimized analogues, for treatment of SARS-CoV-2 infection in human is recommended.

## Abstract (precis)

The coronavirus (CoV) disease 2019 (COVID-19) pandemic caused by severe acute respiratory syndrome CoV-2 (SARS-CoV-2) is a health threat worldwide. Viral main protease (M^pro^, also called 3C‐like protease [3CL^pro^]) is a therapeutic target for drug discovery. Herein, we report that GC376, a broad-spectrum inhibitor targeting M^pro^ in the picornavirus-like supercluster, is a potent inhibitor for the M^pro^ encoded by SARS-CoV-2, with a half-maximum inhibitory concentration (IC_50_) of 26.

## INTRODUCTION

Coronavirus (CoV) infection in humans and other animals has resulted in a variety of highly prevalent and serious diseases, including severe acute respiratory syndrome (SARS) and Middle East respiratory syndrome (MERS). Since late 2019, novel coronavirus disease 2019 (COVID-19), caused by severe acute respiratory syndrome CoV-2 (SARS-CoV-2), has spread from Wuhan City in China to the whole world (1, 2). As with SARS-CoV and MERS-CoV, the newly identified SARS-CoV-2 belongs to the genus *Betacoronavirus* and has a zoonotic origin (3, 4). SARS-CoV-2 causes common symptoms, including fever, cough, and shortness of breath. Complications may include pneumonia and acute respiratory distress syndrome (5, 6).

The genome of COVID-19 virus consists of about 30,000 nucleotides; its replicase gene encodes two overlapping polyproteins, pp1a and pp1ab, which are needed for virus replication and transcription. The functional viral proteins are released from the polypeptide through proteolysis, mainly by the main protease (M^pro^), which is also referred as 3C-like protease (7). M^pro^ can digest at least 11 conserved sites within viral polyproteins. Viral M^pro^ has been considered a therapeutic target for the development of an effective antiviral treatment (8). Among all the mature structural or nonstructural proteins in SARS-CoV-2, M^pro^ is the most conserved target region within the whole viral genome (9). Due to the severity of SARS-CoV-2 infection, it is important to emphasize drug discovery for SARS-CoV-2 based on existing drugs for immediate uses or an expedited development timeline.

We previously discovered several small-molecule inhibitors for SARS-CoV during the SARS outbreak in 2003 (10). The M^pro^ encoded by SARS-CoV-2 represents a key target for anti-SARS-CoV-2 strategies. However, to date, a promising SARS-CoV-2 M^pro^ protease inhibitor has been lacking. Herein, we establish the SARS-CoV-2 M^pro^ protease fluorescence-based assay to screen for potential inhibitors. Furthermore, molecular modeling studies were carried out to further demonstrate the interaction of M^pro^ with GC376. GC376 or its optimized analogues hold great promise to be developed in humans with SARS-CoV-2 infection, alone or together with other antiviral drugs.

## RESULTS

### Fluorescence resonance energy transfer (FRET)-based screening assays.

The M protease (M^pro^) encoded by the SARS and SARS-CoV-2 coronaviruses differ in only 12 amino acid residues. According to our previous experience during the SARS outbreak, SARS-CoV-2 M^pro^ is expressed as a glutathione *S*-transferase (GST) fusion protein in Escherichia coli (11, 12). The GST fusion protein was purified by glutathione affinity chromatography. The fusion protein was cleaved by factor Xa, resulting in the generation of a prominent protein band with an apparent molecular mass of 35 kDa, the mature SARS-CoV-2 M^pro^ (see Fig. S1c in the supplemental material).

Purified M^pro^ proteins were checked for the proteolytic activity that cleaves the EDANS-KTSAVLQSGFRKME-DABCYL substrate, where EDANS is 5-((2-aminoethyl)amino)naphthalene-1-sulfonic acid and DABCYL is 4-(dimethylaminoazo)benzene-4-carboxylic acid (Fig. S2a). The purified enzyme was assayed using the FRET technique as described in Materials and Methods. The performance of the FRET assay was assessed, and the signal-to-noise ratio was determined to be >20 (Fig. S2b). The Z-factor value for the assay was 0.9, which corresponds to a valid screening system. The M^pro^ of feline infectious peritonitis virus (FIPV) was prepared similarly. The same fluorogenic substrate, EDANS-KTSAVLQSGFRKME-DABCYL, was equally applicable in activity assessments for FIPV M^pro^.

### Inhibitory activities of SARS-CoV-2 M^pro^ by the Zinc ion, GC376, and lopinavir.

**FIG 1:**
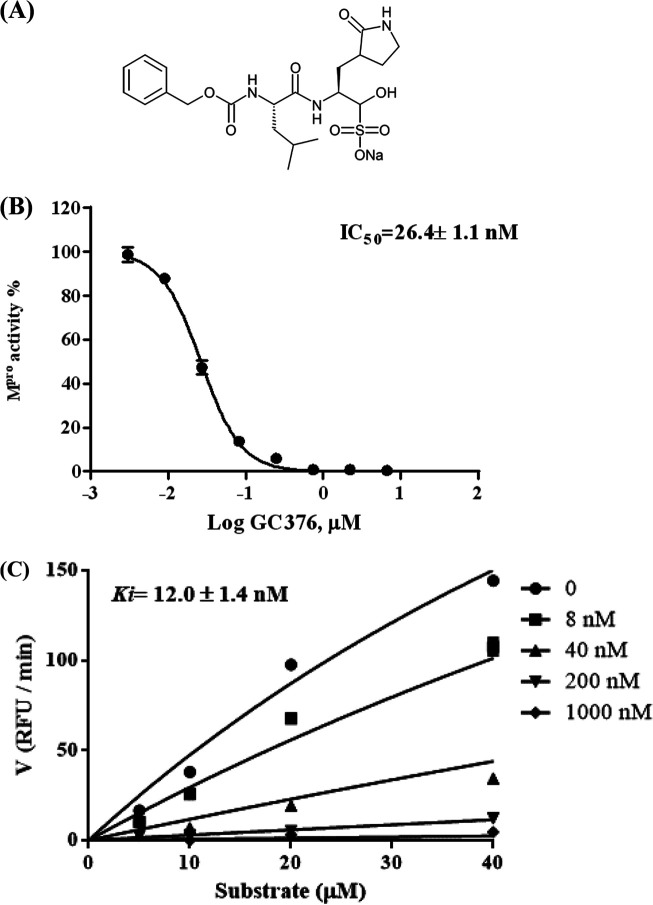
Structure of GC376 and the IC_50_ and the inhibitory constant (*K_i_*) of recombinant M^pro^ of SARS-CoV-2. (A) GC376 is a peptidomimetic antiviral drug. The IC_50_ (B) and the *K_i_* (C) of M^pro^ of SARS-CoV-2 are shown. The proteolytic activity of M^pro^ was determined by the FRET protease assay, as described in the text. RFU, relative fluorescence units.

In this study, based on the SARS-CoV-2 M^pro^ activity assay, we screened a collection of protease inhibitors, such as reported M^pro^ inhibitors for relevant viruses and clinically approved human immunodeficiency virus (HIV) protease inhibitors. At 10 μM, GC376 (Fig. 1A), a broad-spectrum antiviral protease inhibitor used to treat cats with FIPV infection (13), showed complete inhibition of SARS-CoV-2 M^pro^ activity. Since GC376 was well characterized for its inhibition of M^pro^ encoded by FIPV, we conducted a head-to-head comparison for the inhibitory activity of GC376 on SARS-CoV-2 M^pro^ and FIPV M^pro^. SARS-CoV-2 and SARS-CoV share high identity in amino acid sequence (Fig. S3a), whereas SARS-CoV-2 M^pro^ and FIPV M^pro^ share 45% identity in amino acid sequence (Fig. S3b). These two viral proteases also share similar folding and crystal structures (14, 15). In this study, we found that GC376 is an extremely potent inhibitor of the M^pro^ encoded by SARS-CoV-2, with a half-maximum inhibitory concentration (IC_50_) of 26.4 ± 1.1 nM (Fig. 1B). Subsequent analysis showed that GC376 is a competitive inhibitor of the M^pro^ from SARS-CoV-2, with a binding constant (*K_i_*) of 12 ± 1.4 nM (Fig. 1C). In contrast, the IC_50_ and *K_i_* of GC376 toward FIPV M^pro^ are 118.9 ± 1.1 nM and 42.5 ± 2.9 nM, respectively (Fig. S4a and b).

To examine whether a covalent adduct is formed, SARS-CoV-2 M^pro^ incubated with GC376 was subject to mass spectrometry (MS) analysis in accordance with a method described previously. Indeed, a gain of 403.2 Da in mass was observed in new peaks from GC376-incubated SARS-CoV-2 M^pro^, indicating the same mechanism for adduct formation as described previously (13; also results not shown). Through the mass spectrometry analysis, we observed a new MS peak with a mass value of 34,194.0 Da, which is equal to the dihydrogen molecular weight of M protease (33,790.8), reflecting conjugation with only one GC376 molecule. Even in an excess of GC376, only 30% of the M^pro^ enzyme was conjugated based on the peak intensity. With the X-ray (NCBI Protein Data Bank accession number 7BRR; release date, 13 May 2020) and the MS analyses performed in this study, it is evident that GC376 forms a covalent bond with Cys145 of M^pro^. That only a small portion of SARS-CoV-2 M^pro^ was covalently modified in a 25:1 molar excess of GC376 indicates that improved inhibitors are needed.

All the HIV protease inhibitors, including lopinavir, ritonavir, fosamprenavir, saquinavir, nelfinavir, atazanavir, darunavir, amprenavir, tipranavir, and indinavir, showed no inhibitory activity at 20 μM, reflecting the fact that no benefit was observed with lopinavir-ritonavir treatment in patients with severe COVID-19 (16). Since Zn^2+^ was shown to inhibit 3CL^pro^ encoded by SARS-CoV (17), ZnCl_2_ and ZnSO_4_ were evaluated for their activity against SARS-CoV-2 M^pro^ in this study. Zinc salts have been shown to completely inhibit the activity of SARS-CoV-2 M^pro^ at the micromolar level (data not shown).

### Antiviral effects of GC376 on the replication of SARS-CoV-2 in cell culture.

**FIG 2:**
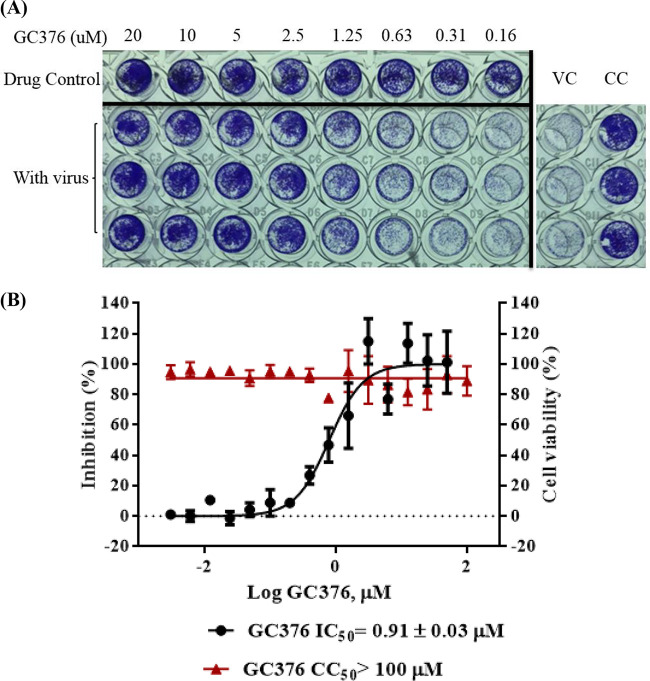
GC376 inhibited SARS-CoV-2 virus replication in Vero E6 cells. (A) Inhibition of SARS-CoV-2-induced CPE by GC376. In a 96-well plate, Vero E6 cells were infected with SARS-CoV-2 virus (100 TCID_50_ per well), and cells were treated with various concentrations of GC376. At 120 h postinfection (hpi), cells were examined with a microscope (magnification, ×100). The cell control (CC) column refers to cells without compound treatment and virus infection. Vero E6 cells were all lysed at 120 h after SARS-CoV-2 infection as shown in the virus control (VC) column. GC376 was added to SARS-CoV-2-infected cells in twofold serial dilutions starting from a concentration of 20 μM in the left-hand column. The drug control (DC) column refers to cells in the absence or presence of GC376 without virus infection. Cells were fixed with formaldehyde and stained with 0.1% crystal violet. Results from one representative plate of two are shown. (B) Effects of GC376 on SARS-CoV-2-induced CPE or cell proliferation were generated using a sigmoidal dose-response curve model (GraphPad Prism 6 software) from which the IC_50_ values were derived. The effect of GC376 on cell proliferation was determined by MTT assay.

To confirm that GC376 inhibited SARS-CoV-2 replication and cellular toxicity in cell culture, GC376 was tested for inhibition of SARS-CoV-2 infection in Vero E6 cells with 100 50% tissue culture infectious doses (TCID_50_) per well in 96-well plates. SARS-CoV-2-infected cells were treated with increasing concentrations of GC376, and protection from cytopathic effects (CPE) was visually observed. GC376 dose dependently showed a reduction of the viral CPE (Fig. S5). After the cells were stained with crystal violet, their optical density at 570 nm (OD_570_) was measured (Fig. 2A). The results showed that GC376 inhibited SARS-CoV-2 infection, with an EC_50_ of 0.91 ± 0.03 μM (Fig. 2B). GC376 exhibited a broad-spectrum antiviral activity against several coronaviruses in various cell lines (13). To evaluate whether GC376 was cytotoxic to cells, Vero E6 cells were treated with different concentrations of GC376 up to 100 μM, and cell viability was determined using the 3-(4,5-dimethyl-2-thiazolyl)-2,5-diphenyl-2H-tetrazolium bromide (MTT) assay. We found that GC376 did not show cytotoxicity in Vero E6 cells up to 100 μM (Fig. 2B). Hence, we concluded that the selectivity index (SI) of GC376 was >114.

### Molecular docking.

**FIG 3:**
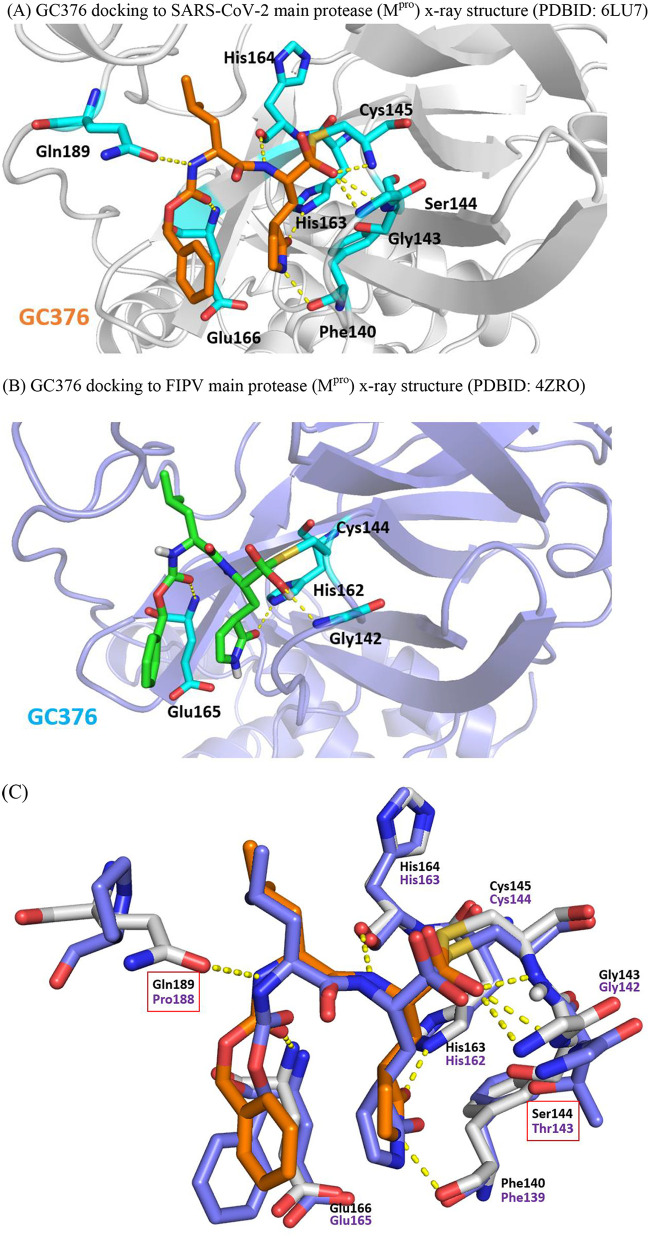
Docked conformations of GC376 in SARS-CoV-2 and FIPV M^pro^ proteases. (A) GC376 docking to the SARS-CoV-2 M^pro^ protein X-ray structure (NCBI Protein Data Bank accession no. 6LU7). (B) GC376 docking to the FLIP 3C-like protease protein X-ray structure (accession no. 4ZRO). (C) Structure alignment of FIPV (accession no. 4ZRO, purple) and SARS-CoV-2 (accession no. 6LU7, white). The docking result of GC376 in FIPV displays as cyan, and the docking result of GC376 displays as orange. The red box shows the different residues in the FIPV and SARS-CoV-2 binding sites.

To inform lead optimization efforts starting from GC376, *in silico* calculations to correlate IC_50_ and *K_i_* into binding energy between GC376 and M^pro^ were attempted in accordance with, in part, our previous work (18–20). By our calculation, the free binding energies of GC376 with SARS-CoV-2 M^pro^ and FIPV M^pro^ are −51.59 kcal/mol and −32.42 kcal/mol, respectively. As shown in Fig. 3A and B, upon removal of the bisulfite group, the compound is converted to an aldehyde form, giving rise to a covalent bond with catalytic Cys145. This result is in congruence with the cocrystal structures of GC376 and MERS M^pro^, where GC376 forms a covalent bond with Cys148 (31). In Fig. 3C, the amino acid residues on the inner surface of the substrate binding sites within FIPV M^pro^ and SARS-CoV-2 M^pro^ are well conserved. Only two sites of amino acid residues are different between the two M^pro^ binding pockets. In SARS-CoV-2, the Gln189 on the surface of the binding pocket of SARS-CoV-2 M^pro^ supports a H bond with the carbamate moiety of GC376. In contrast, this H bond cannot be formed because the counterpart residue in FIPV M^pro^ is Pro188, rather than Gln. It appears that due to the covalent binding with Cys145 and hydrogen binding with Gln189 within the substrate binding pocket of SARS-CoV-2 M^pro^, GC376 was induced to bind more snugly into the pocket through a strong H bond network with Phe140, Gly143, Ser144, Cys145, His163, His164, Glu166, and Gln189 (Fig. 3A). In contrast, GC376 forms only a weaker H bond network with Gly142, His162, and Glu165 (Fig. 3B). The other different sites are Ser144 in SARS-CoV-2 M^pro^ and Thr143 in FIPV M^pro^. These differences had little influence on the binding network. The root mean square deviation (RMSD) between the two docking conformations of GC376/SARS-CoV-2 and GC376/FIPV is 1.16 Å. Importantly, our *in silico* prediction has informed our potential direction to improve GC376 with respect to its potency and further drug-like properties. With the docking analyses, GC376 may be improved by replacing the benzene group with H bond donors to interact with Glu166. The other alternative for improvement of binding potency is to replace the isobutyl group with moieties of a less bulky hydrophobic group so as to form interactions with Met49 (Fig. 3C). R. J. Hussey et al. have reported that the Michael acceptor inhibitor, acetyl-Glu-Phe-Gln-Leu-Gln-CH=CHCOO–, forms a covalent bond with catalytic Cys139 in norovirus 3CL^pro^ (22), suggesting an alternative avenue for optimization of GC376. After the submission of this paper, the crystal structure of the 3CL protease complexed with GC376 (accession no. 7BRR; release date, 13 May 2020) became available in the NCBI Protein Data Bank. When we compared the cocrystal X ray with our docked M^pro^-GC376 model based on 6LU7, the RMSD was 0.74, indicating that the *in silico* docking approach employed in this study adequately predicted the real complexed structure before its availability. In Fig. S6, the modeled conformation is shown in green and aligned to the X-ray result in pink.

## DISCUSSION

To date, no proven effective therapy has been shown to be effective for SARS-CoV-2 infection (9). As of the submission date of this paper, the once-promising medicines, including remdesivir and hydroxychloroquine, are facing challenges after more stringently controlled observations and trials (23, 24). When coronaviruses replicated inside cells, cellular innate immunity was shown to be compromised by M^pro^ (25). We have previously shown that the compromised interferon (IFN)-mediated antiviral mechanism of viral 3C^pro^ of enterovirus 71 can be rescued by effective protease inhibitor (26). Thus, effective inhibition of viral protease may not only restrict virus replication but also prevent interruption of the antiviral IFN pathway.

We also found that GC376 is a promising M^pro^ inhibitor for SARS-CoV-2. GC376 is a dipeptidyl bisulfite adduct salt with excellent inhibitory activity against several picornaviruses and coronaviruses (13, 27, 28). Administration of GC376 leads to a full recovery in laboratory cats with FIPV infection, a highly fatal feline disease (29). Y. Kim in 2016 also studied the pharmacokinetic properties and the safety of GC376 in laboratory cats. In their safety study of GC376, no adverse effects were observed and no changes in clinical lab parameters were reported in cats subcutaneously given GC376 at 10 mg/kg of body weight/dose twice a day for 4 weeks (29). In this safety study, the plasma drug concentrations were shown to remain slightly above 1,000 ng/ml (i.e., ∼2,000 nM, as the molecular weight of GC376 is 507.53), which was well above the concentrations needed for effective inhibition of SARS-CoV-2 as observed in this study. Therefore, the existing pharmacology and efficacy data for GC376 as an investigational drug in cats with FIPV infection encourage a proof-of-principle study of COVID-19 patients and then of the *in vitro* and *in vivo* antiviral activities of GC376 or further-optimized analogues.

## MATERIALS AND METHODS

### Drugs and reagents.

The test compounds were mainly from Selleck and MedChemExpress. Several in-house-collected compounds, including HIV protease inhibitors, GC376, and natural products, were also screened as M^pro^ inhibitors. GC376 was purchased from Biosynth Carbosynth. It was dissolved in dimethyl sulfoxide (DMSO) as a 10 mM stock solution and stored at −20°C. The fluorogenic peptide substrate DABCYL-KTSAVLQSGFRKME-EDANS utilized in the fluorescence resonance energy transfer (FRET) assay for M^pro^ was obtained from Genesis Biotechnology Inc.

### Expression and purification of 3C-like proteases (M^pro^).

To express the M proteases from SAR-CoV-2 and FIPV, cDNAs encoding factor Xa cleavage site and the genes as deduced from the Wuhan-Hu-1 strain (GenBank accession no. NC_045512.2) and WSU-79/1146 strain (NCBI accession no. AAY32595.1) were optimized for codon preference in E. coli, respectively. The amino acid sequence of SARS-CoV-2 M^pro^ is shown in Fig. S1a in the supplemental material. The synthetic cDNA (Bio Basic, Canada) encoding factor Xa recognition site and M^pro^ was inserted into the expression plasmid vector pGEX-4T-1 (GE Health Care) using BamHI and XhoI restriction enzyme cutting sites (Fig. S1b); an ampicillin resistance gene was used as a selection marker.

The recombinant plasmid was transfected into the Rosetta 2(DE3)pLysS strain (Novagen) and an E. coli host, and the overnight culture in LB medium was refreshed to an OD_600_ of 0.8 at 37°C and then induced with 1 mM IPTG for 5 h at 25°C. The cells were harvested by centrifugation at 4°C (6,000 rpm, 10 min) followed by sonication in lysis buffer containing 1× phosphate-buffered saline (137 mM NaCl, 10 mM phosphate, 2.7 mM KCl, pH 7.4), 0.1% Triton X-100. The GST-M^pro^ fusion protease was purified by Glutathione Sepharose 4 Fast Flow (GE Healthcare) with purification buffer (50 mM Tris, pH 8.0, 10 mM glutathione). The purified GST-M^pro^ fusion protease was changed to factor Xa digestion buffer (20 mM Tris, pH 6.6, 50 mM NaCl, 1 mM CaCl_2_) and digested with factor Xa at 20°C overnight. The digested GST-M^pro^ was reloaded into a glutathione Sepharose column to collect the flowthrough for separation of M^pro^. SDS-PAGE analysis shows that M^pro^ is purified with approximately 95% purity. M^pro^ was moved to storage buffer (50 mM Tris, 100 mM NaCl, 1 mM KCl, 1 mM CaCl_2_, 25% glycerol) using an Amicon filter (10,000 cutoff; Millipore), aliquoted, and stored at –20°C.

### Protease activity assay.

The protease assays were performed in 96-well, black, flat-bottomed microtiter plates (Greiner Bio-One, Germany) with a final volume of 100 μl. SARS-CoV-2 M^pro^ recombinant protease, at a final concentration of 20 nM, was preincubated for 10 min at room temperature (RT) with compounds at different concentrations in the assay buffer (20 mM HEPES, pH 6.0, 0.4 mM EDTA, 1 mM dithiothreitol [DTT], 1% glycerol). The FRET substrate, DABCYL-KTSAVLQSGFRKME-EDANS, was then added at a final concentration of 10 μM to the enzymatic reaction mixture for 30 min at RT. The readouts for the same compound concentrations with the substrate without M^pro^ enzyme were measured as a blank. The fluorescence signals (excitation/emission, 355 nm/460 nm) of released EDANS were measured using a fluorometer (Victor2; PerkinElmer). The results were plotted as dose inhibition curves using nonlinear regression with a variable slope to determine the IC_50_ values of inhibitor compounds (with GraphPad Prism 5.0). *K_i_* measurements were performed with various substrate concentrations of 5, 10, 20, and 40 μM and with a range of inhibitor concentrations (0, 8, 40, 200, and 1,000 nM) in a reaction mixture containing 20 nM M^pro^ at 37°C for 30 min. The *K_i_* value was computed using GraphPad Prism 5.0 software by nonlinear regression of competitive enzyme kinetics.

### Antiviral assay and cytotoxicity assay.

To examine the anti-SARS-CoV-2 activity of positive compounds identified in the M^pro^ activity assay, TCID_50_ (50% tissue culture infectious doses) were determined using 2-fold serial dilutions of hit compounds starting from 50 μM. In brief, each well of a 96-well tissue culture plate was seeded with 200 μl of 1.15 × 10^5^ Vero E6 cells/ml in minimal essential medium (MEM) with 10% fetal bovine serum (FBS). After cells were incubated for 18 to 24 h at 37°C, SARS-CoV-2/human/TWN/CGMH-CGU-01/2020 virus was added at 100× TCID_50_ per well mixed with different concentrations of GC376. After 5 days, cells were fixed with formaldehyde and stained with 0.1% crystal violet as described previously (21). The concentration required for the tested compound to reduce the cytopathic effects (CPE) of the virus by 50% (the 50% effective concentration [EC_50_]) was determined. The IC_50_ was calculated using GraphPad Prism 6 to assess the inhibition percentage at different inhibitor concentrations. To estimate the safety profile, an *in vitro* cytotoxicity study of GC376 was performed. We used the MTT assay to investigate the cytotoxicities of these compounds on Vero E6 cells. The half of the cytotoxic concentration (CC_50_) values were calculated from the percentages of cells whose viability was inhibited by GC376 at various concentrations.

### Molecular modeling.

The docking of compounds to the binding sites of SARS-CoV-2 M^pro^ (NCBI Protein Data Bank accession no. 6LU7) (14) or M^pro^ of feline infectious peritonitis virus, FIPV M^pro^ (NCBI Protein Data Bank accession no. 4ZRO) (6), was explored using the BIOVIA 2018/LigandFit program (BIOVIA, Inc., San Diego, CA). The detailed method of LigandFit has been described previously (30). To illustrate the binding interactions, GC376 was docked to the binding site. The binding pocket was identified from the MERS and GC376 cocrystal structures (NCBI Protein Data Bank accession no. 5WKJ) (31). The force field for calculating ligand-receptor interaction energies employed the piecewise linear potential 1 (PLP1). The rectangular grid was set at 0.5 Å spacing, and the extension from the site was set as 8 Å. The number of docking poses was set as 50 with default parameters. The docking root mean square (RMS) threshold for ligand site matching was set as 5 Å. The method of steepest descent for rigid-body minimization during pose docking was used. The covalent docking calculation was performed using the two-point attractor method by the AutoDock Tools (version 1.5.6) as described previously (17). The decision of the best pose was based on the similar conformations of the MERS complex cocrystal structure.

### Molecular weight analysis using MS.

The premixed GC376 compound (1 μl, 10 mM in DMSO) and SARS-CoV-2 M^pro^ (5 μl, 2.5 mg/ml) were incubated at 25°C for 30 min. Subsequently, 10 μl of the reaction mixture was transferred into 40 μl of the infusion solution (50% acetonitrile in 0.1% formic acid) for measuring the molecular weight using a quadrupole time of flight (QTOF) mass spectrometer (G1; Waters) through the direct infusion model. The ion signal (*m/z*) was acquired in positive-ion mode with a capillary temperature of 100°C and an electrospray voltage of 2,800 V in the scan range from 800 to 2,500 *m/z*. Mass deconvolution was performed using Waters MassLynx (v4.1) software using the MaxEnt 1 program with a half-height of 0.1 Da and a maximum number of interactions of 100.

## Supplementary Material

Supplemental file 1
