# Supplementary material for: Discovery of M Protease Inhibitors Encoded by SARS-CoV-2
Source: Antimicrob Agents Chemother. 2020 Aug 20;64(9):e00872-20. doi: 10.1128/AAC.00872-20 (PMC7449189; doi:10.1128/AAC.00872-20)
Supplement: Supplemental file 1 [file AAC.00872-20-s0001.pdf]

Supplementary Data

1.

(a)

```
            S  G  F  R  K  M  A  F  P  S  G  K  V  E  G  C  M  V  Q  V
  1  AGCGGCTTCCGT AAGATGGCGTTT CCGAGCGGCAAA GTTGAAGGTTGC ATGGTGCAGGTT
            T  C  G  T  T  T  L  N  G  L  W  L  D  D  V  V  Y  C  P  R
 61  ACCTGCGGCACC ACCACCCTGAAC GGTCTGTGGCTG GACGATGTGGTT TACTGCCCGCGT
            H  V  I  C  T  S  E  D  M  L  N  P  N  Y  E  D  L  L  I  R
121  CACGTTATCTGC ACCAGCGAGGAC ATGCTGAACCCG AACTATGAGGAC CTGCTGATTCGT
            K  S  N  H  N  F  L  V  Q  A  G  N  V  Q  L  R  V  I  G  H
181  AAGAGCAACCAC AACTTCCTGGTG CAGGCGGGCAAC GTGCAACTGCGT GTTATCGGTCAC
            S  M  Q  N  C  V  L  K  L  K  V  D  T  A  N  P  K  T  P  K
241  AGCATGCAGAAC TGCGTGCTGAAG CTGAAAGTTGAC ACCGCGAACCCG AAAACCCCGAAG
            Y  K  F  V  R  I  Q  P  G  Q  T  F  S  V  L  A  C  Y  N  G
301  TACAAATTCGTG CGTATTCAGCCG GGCCAAACCTTT AGCGTTCTGGCG TGCTACAACGGC
            S  P  S  G  V  Y  Q  C  A  M  R  P  N  F  T  I  K  G  S  F
361  AGCCCGAGCGGT GTTTATCAATGC GCGATGCGTCCG AACTTCACCATC AAGGGTAGCTTT
            L  N  G  S  C  G  S  V  G  F  N  I  D  Y  D  C  V  S  F  C
421  CTGAACGGTAGC TGCGGCAGCGTG GGTTTCAACATT GACTACGATTGC GTTAGCTTTTGC
            Y  M  H  H  M  E  L  P  T  G  V  H  A  G  T  D  L  E  G  N
481  TATATGCACCAC ATGGAGCTGCCG ACCGGCGTTCAT GCGGGCACCGAC CTGGAGGGCAAC
            F  Y  G  P  F  V  D  R  Q  T  A  Q  A  A  G  T  D  T  T  I
541  TTCTACGGTCCG TTTGTGGACCGT CAAACCGCGCAA GCGGCGGGCACC GATACCACCATC
            T  V  N  V  L  A  W  L  Y  A  A  V  I  N  G  D  R  W  F  L
601  ACCGTGAACGTT CTGGCGTGGCTG TATGCGGCGGTG ATTAACGGTGAC CGTTGGTTCCTG
            N  R  F  T  T  T  L  N  D  F  N  L  V  A  M  K  Y  N  Y  E
661  AACCGTTTTACC ACCACCCTGAAC GATTTCAACCTG GTGGCGATGAAG TACAACTATGAG
            P  L  T  Q  D  H  V  D  I  L  G  P  L  S  A  Q  T  G  I  A
721  CCGCTGACCCAA GATCATGTGGAC ATCCTGGGTCCG CTGAGCGCGCAA ACCGGTATTGCG
            V  L  D  M  C  A  S  L  K  E  L  L  Q  N  G  M  N  G  R  T
781  GTGCTGGACATG TGCGCGAGCCTG AAAGAACTGCTG CAAAACGGCATG AACGGTCGTACC
            I  L  G  S  A  L  L  E  D  E  F  T  P  F  D  V  V  R  Q  C
841  ATTCTGGGCAGC GCGCTGCTGGAG GACGAGTTCACC CCGTTTGATGTG GTTCGTCAGTGC
            S  G  V  T  F  Q
901  AGCGGTGTGACC TTTCAA
```

SGFRKMAFPSGKVEGCMVQVTCGTTTLNGLWLDDVVYCPRHVICTSEDMLNPN
YEDLLIRKSNHNFLVQAGNVQLRVIGHSMQNCVLKLKVDTANPKTPKYKFVRIQ
PGQTFSVLACYNGSPSGVYQCAMRPNFTIKGSFLNGSCGSVGFNIDYDCVSFCY
MHHMELPTGVHAGTDLEGNFYGPFVDRQTAQAAGTDTTITVNVLAWLYAAVIN
GDRWFLNRFTTTLNDFNLVAMKYNYEPLTQDHVDILGPLSAQTGIAVLDMCASL
KELLQNGMNGRTILGSALLEDEFTPFDVVRQCSGVTFQ

(b).

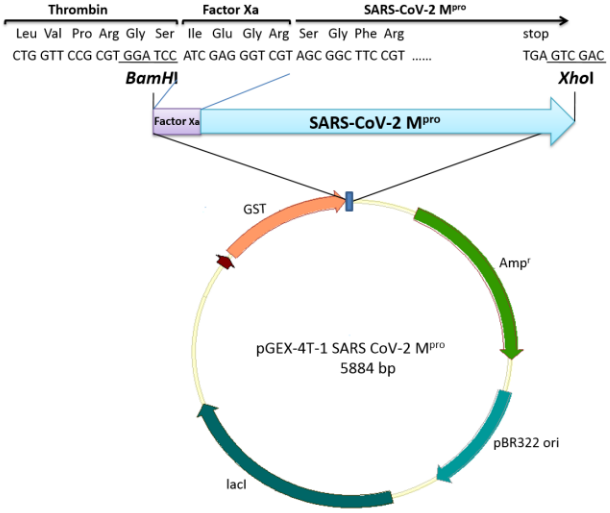
Thrombin
Factor Xa
SARS-CoV-2 Mpro
Leu Val Pro Arg Gly Ser Ile Glu Gly Arg Ser Gly Phe Arg stop
CTG GTT CCG CGT GGA TCC ATC GAG GGT CGT AGC GGC TTC CGT ...... TGA GTC GAC
BamHI
XhoI
Factor Xa
SARS-CoV-2 Mpro
GST
Ampr
pGEX-4T-1 SARS CoV-2 Mpro
5884 bp
pBR322 ori
lacI

(c).

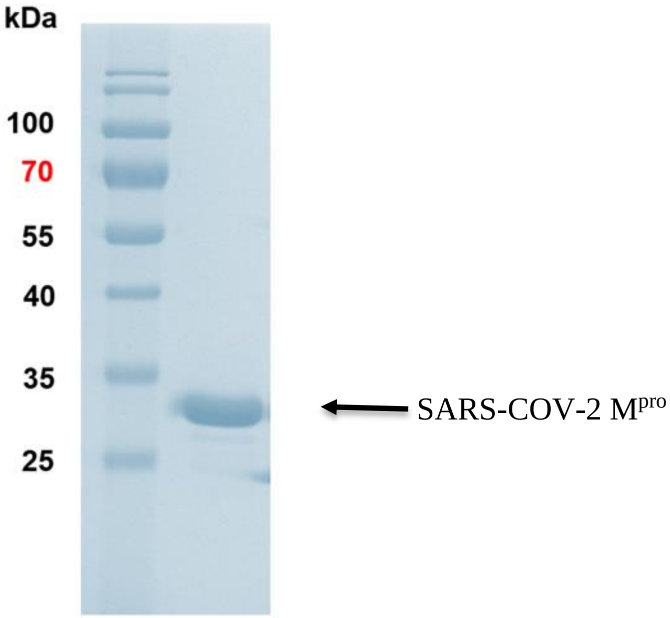
kDa
100
70
55
40
35
25
SARS-COV-2 Mpro

2.

(a)

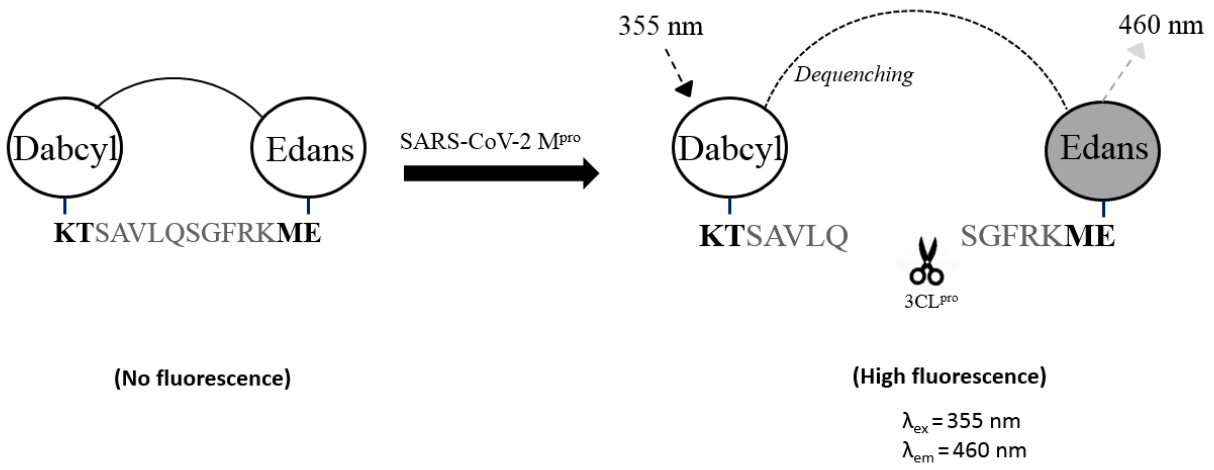
355 nm
460 nm
Dequenching
Dabcyl
Edans
KTSAVLQSGFRKME
SARS-CoV-2 Mpro
Dabcyl
Edans
KTSAVLQ
SGFRKME
3CLpro
(No fluorescence)
(High fluorescence)
λex = 355 nm
λem = 460 nm

(b)

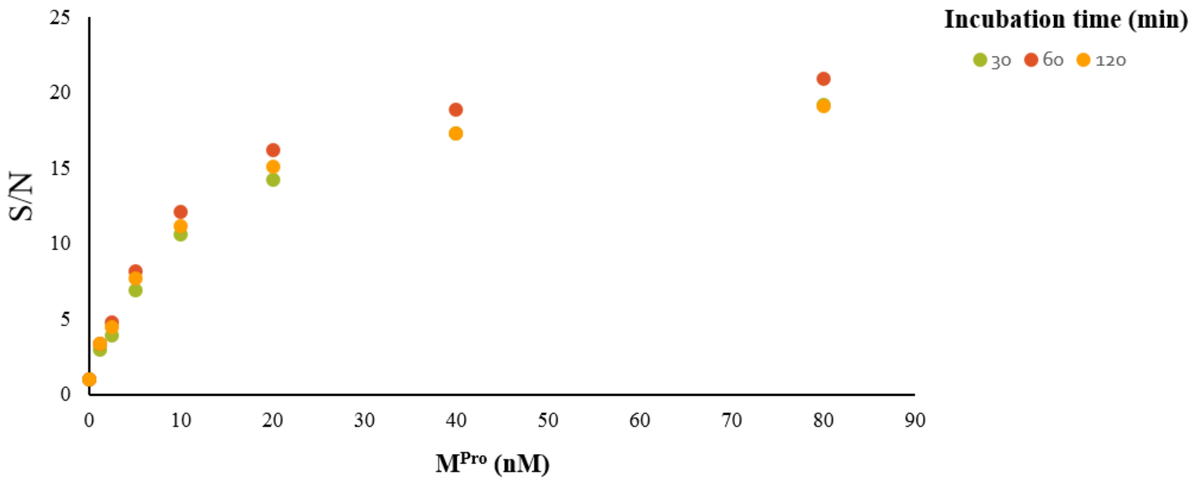
Incubation time (min)
30
60
120
S/N
25
20
15
10
5
0
0
10
20
30
40
50
60
70
80
90
MPro (nM)

3

(a) Protein Sequence Alignment: SARS-CoV M^{pro} with SARS-CoV-2 M^{pro}

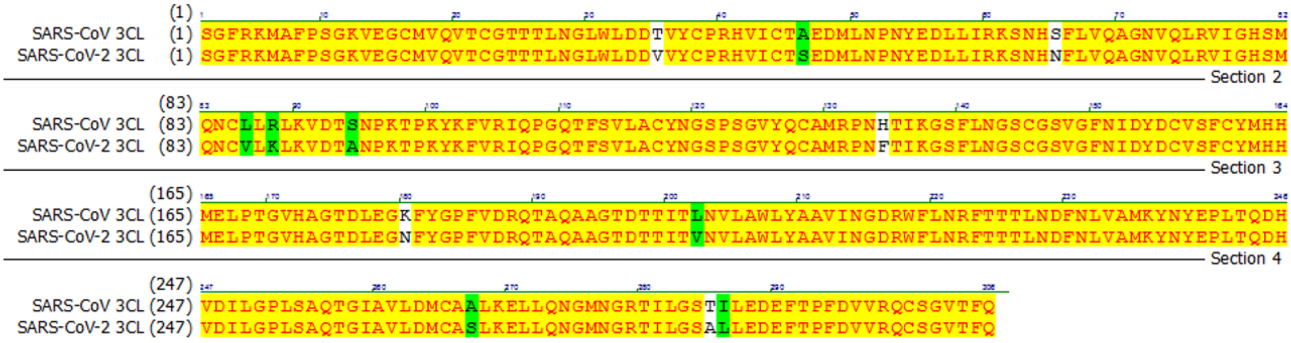

(b) Protein Sequence Alignment: FIPV-M^{pro} with SARS-CoV-2 M^{pro}

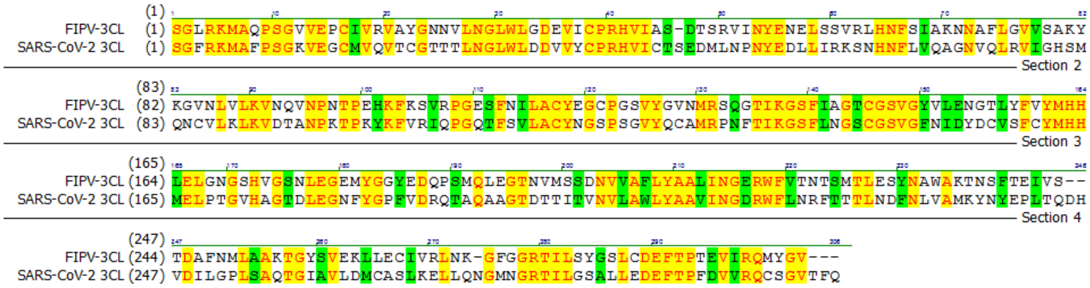

4.

(a)

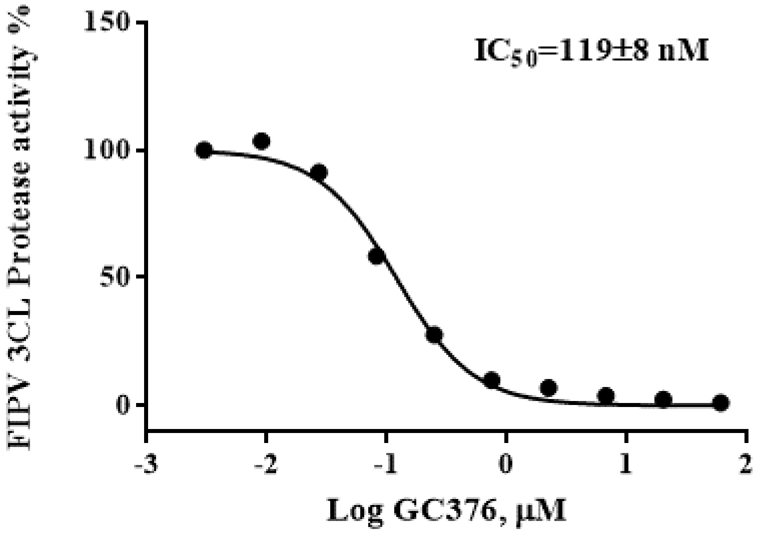
IC50=119±8 nM
FIPV 3CL Protease activity %
Log GC376, μM
150
100
50
0
-3
-2
-1
0
1
2

(b)

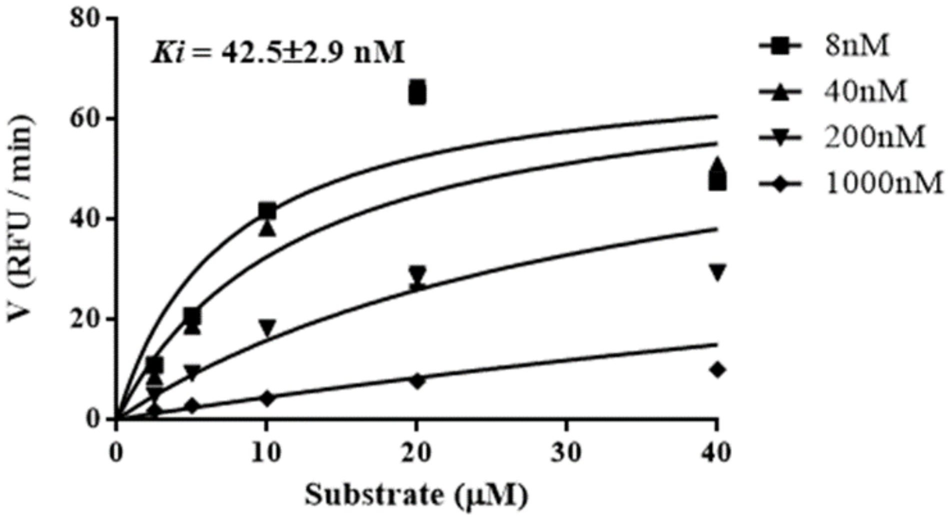
Ki = 42.5±2.9 nM
V (RFU / min)
Substrate (μM)
8nM
40nM
200nM
1000nM
80
60
40
20
0
0
10
20
30
40

5.

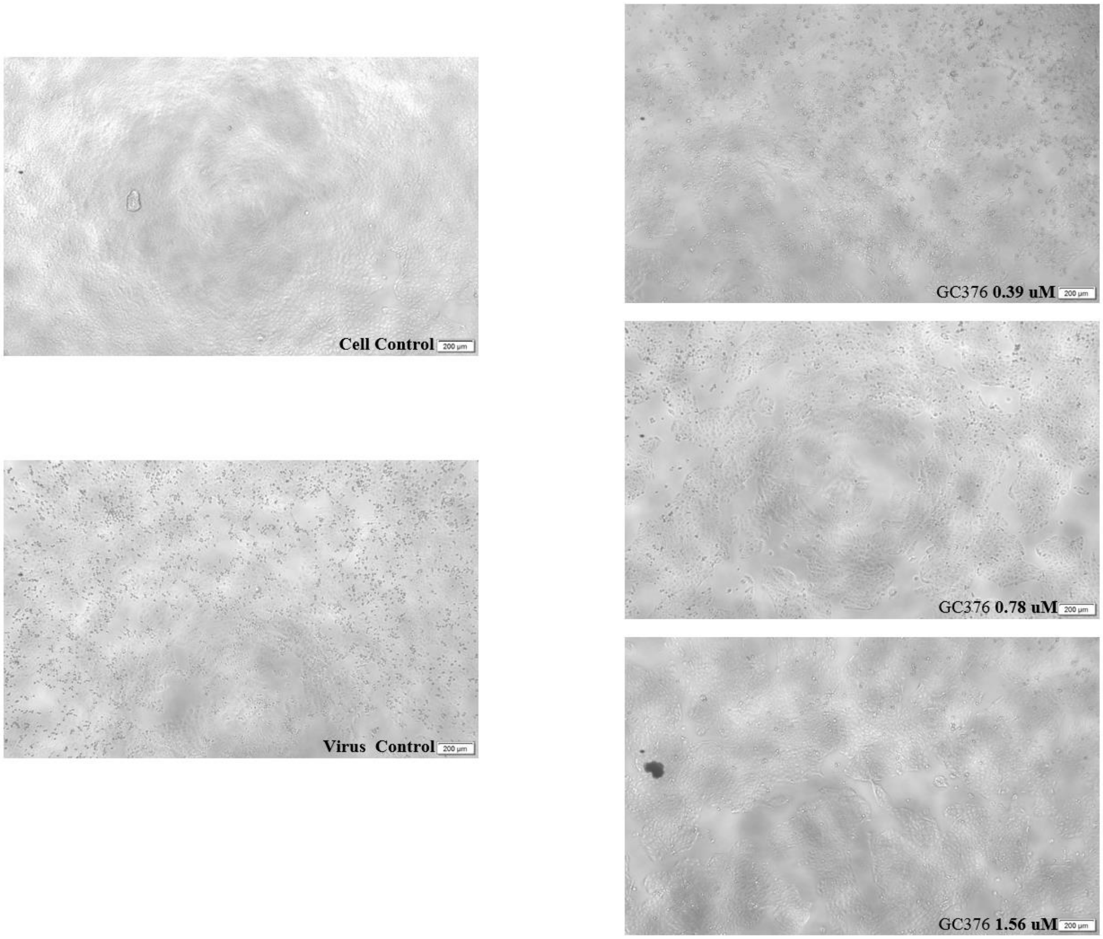
Cell Control
Virus Control
GC376 0.39 uM
GC376 0.78 uM
GC376 1.56 uM

6.

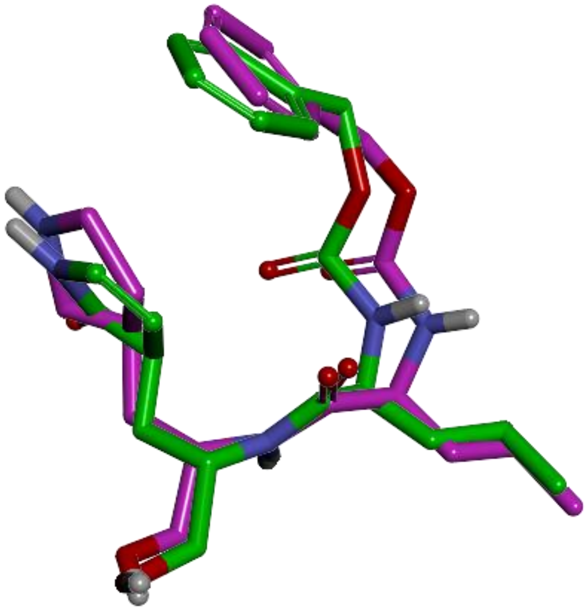

Supplementary Data Legends

S1. SARS-CoV-2 M^{pro} plasmid construction and protein expression.

(a) The amino acid sequence of the M^{pro} from Wuhan-Hu-1 strain (NC_045512.2) is as shown. (b) The synthetic cDNA (BIO BASIC, Canada) was inserted into expression plasmid vector pGEX-4T-1 (GE Health Care) using *BamH* I and *Xho* I restriction enzyme cutting sites. (c) Purified SARS-CoV-2 M^{pro} was resolved with SDS-PAGE under reducing conditions and visualized by Coomassie Blue staining, showing an apparent molecular mass of approximate 35 kDa.

S2. Development SARS-CoV-2 M^{pro} FRET assay to facilitate enzyme assay and inhibitor screening.

(a) Schematic diagram illustrating the principle of the FRET assay. The autocleavage sequence SAVLQSGFRK of M^{pro} is inserted between the fluorescence quenching pair (Edans-Dabcyl) in the fluorogenic peptide substrate for the SARS-CoV-2 M^{pro} inhibition assay. Cleavage of the peptide bond at SAVLQ ↓ SGFRK by SARS-CoV-2 M^{pro} proteins separates Dabcyl and Edans, enhanced fluorescence caused by cleavage of the fluorogenic substrate peptide can be monitored at 460 nm with excitation at 355 nm. (b) Recombinant SARS-CoV-2 Mpro was titrated and assayed in 96-well plates using fluorogenic peptide substrate. The fluorescence were monitored after 30 minutes on a fluorometer (VICTOR2, PerkinElmer). The results are plotted as signal-to-noise ratios.

S3. Sequence Alignment of coronaviruses with SARS-CoV-2

(a) The M^{pro} encoded by these SARS and SARS-CoV-2 coronaviruses differ in only 12 amino acid residues. Sequences alignment of SARS and SARS-CoV-2 M^{pro} proteins was performed to identify regions of similarity using Vector NTI ContigExpress software (ThermoFisher Scientific). **(b) The M^{pro} encoded by these FLIP and SARS-CoV-2 coronaviruses.** Sequences alignment of FLIP and SARS-CoV-2 M^{pro} proteins was performed to identify regions of similarity using Vector NTI ContigExpress software (ThermoFisher Scientific).

S4. Inhibition of M^{pro} of FIPV by GC376.

The IC_{50} (a) and the inhibitory constant (Ki) (b) of Recombinant M^{pro} of FIPV were used in the fluorogenic substrate method with various concentrations of GC376.

S5. **The inhibition of SARS-CoV-2-induced CPE by GC376.**

In 96-well plate, Vero E6 cells were infected with SARS-CoV-2 virus (100 $TCID_{50}$ per well) and treated with various concentrations of GC376. At 120 h post-infection (*hpi*), cells were examined by a microscope (100×). Cell control: normal cells without treatment; Virus: cells infected with SARS-CoV-2 virus at 100 $TCID_{50}$/well; in the absence of presence of GC376 at 0.39, 0.78 or 1.56 μM, respectively.

S6. **Comparison of computational model and X-ray crystal structure of M^{pro}-GC376.** The modeling conformation of GC376 (green) in complex with M^{pro} of SARS-CoV-2 aligned to the GC376 (pink) extracted from X-ray of GC376/SARS-CoV-2-M^{pro} cocrystal structure (7BRR).
